# Simulation-Based Prediction of Equivalent Continuous Noises during Construction Processes

**DOI:** 10.3390/ijerph13080818

**Published:** 2016-08-12

**Authors:** Hong Zhang, Yun Pei

**Affiliations:** Institute of Construction Management, Zhejiang University, Zhejiang 310058, China; py255650708@126.com

**Keywords:** equivalent continuous noise, construction processes, prediction, discrete-event simulation, simulation framework

## Abstract

Quantitative prediction of construction noise is crucial to evaluate construction plans to help make decisions to address noise levels. Considering limitations of existing methods for measuring or predicting the construction noise and particularly the equivalent continuous noise level over a period of time, this paper presents a discrete-event simulation method for predicting the construction noise in terms of equivalent continuous level. The noise-calculating models regarding synchronization, propagation and equivalent continuous level are presented. The simulation framework for modeling the noise-affected factors and calculating the equivalent continuous noise by incorporating the noise-calculating models into simulation strategy is proposed. An application study is presented to demonstrate and justify the proposed simulation method in predicting the equivalent continuous noise during construction. The study contributes to provision of a simulation methodology to quantitatively predict the equivalent continuous noise of construction by considering the relevant uncertainties, dynamics and interactions.

## 1. Introduction

Construction processes produce not only gas emission pollution [1], but also acoustic pollution, i.e., noise, which harmfully annoys people [2]. Various categories of noise-related problems have been classified, including noise-induced hearing impairment [3], interference with speech communication, disturbance of rest and sleep, psycho-physiological or mental impacts, and interference with intended activities [4]. Some countries have stipulated noise standards for noise control. While the noise ≤70 dBA is generally believed to not cause hearing impairment in the large majority of people [5], construction processes often produce noises over 85–90 dBA [6]. Because construction of buildings or infrastructures often takes place in congested urban areas, construction noise can be a great annoyance to the nearby people in addition to the workers involved. For example, nearly 18% of all noise complaints in Singapore were pertinent to the noise from construction sites [7]. Some governments have also stipulated related regulations or standards, especially for construction noise [8,9,10].

In consideration of the noise fluctuation over time due to uncertain and dynamic construction environments, instantaneous value of noise cannot comprehensively reflect the actual noise level and its influences on people including nearby residents and construction workers [9]. Hence, equivalent continuous noise over a period of time [5,11], called *L_eq-T_* in this paper, is often used to indicate the actual noise level over time. In fact, some construction noise standards [8,9,10] consider the noise in terms of *L_eq-T_*. For example, the China GB12523 noise standard [10] stipulates that the maximum *L_eq-T_* over 20 min (namely *max*-*L_eq-20m_*) should not exceed 75 dBA and 55 dBA, respectively, for daytime and nighttime, while the U.S. noise standard [8] stipulates that the maximum *L_eq-T_* over 8 h (namely *max*-*L_eq-8h_*) at a residential area should not exceed 80 dBA and 70 dBA, respectively, for daytime and nighttime. The instantaneous noise level may be affected by equipment properties and geometric parameters, while the *L_eq-T_* depends on the proportion of time during which multiple equipment items operate concurrently and the number of concurrently operated pieces of equipment. Meanwhile, the proportion of time and the number of concurrently operated equipment are affected by the start time, duration and preceding-logical conditions and resource requirements of each activity. Obviously, there exist complexities such as uncertainties, dynamics and interactions in determining the *L_eq-T_*.

To evaluate construction plans to address noise standards or decide suitable noise reduction measures, it is necessary to predict the construction noise, particularly the equivalent continuous noise. Due to uncertainties, dynamics and interactions in construction, field measurement of the noise and particularly the *L_eq-T_* may be impractical for every case and the results are inapplicable to every case [12,13]. Manatakis and Skarlatos [14] proposed a statistical model for predicting the noise from construction equipment. Hamoda [5] used the Back-Propagation Neural Network (BPNN) to predict the construction noise by modeling the nonlinear and imprecise relationship between inputs and outputs. Monte Carlo technique had been adopted to predict the construction noise by reflecting the uncertainties [15,16,17]. However, these studies did not consider the equivalent continuous noise (*L_eq-T_*) over a period of time, let alone the uncertainties, dynamics and interactions that are particularly sensitive to the *L_eq-T_*. Moreover, these studies could not model interactions among multiple noise-affected factors, leading to difficulty in analyzing major factors. 

Due to the capabilities in modeling uncertainties, dynamics and interactions of an operational system, discrete-event simulation (DES) that has been successfully used to predict durations, costs and productivities of construction process [18,19,20,21]. DES has also been applied to predict construction noises [22]. However, no more relevant references about DES application in predicting construction noises have been noticed so far. In addition, the existing DES application for predicting construction noises still did not address equivalent continuous noise (*L_eq-T_*). Moreover, the simulation framework regarding how to implement the noise-calculation algorithm through DES was not clearly presented.

With regards to the abovementioned backgrounds, this study focuses on applying the DES method to predict the equivalent continuous noise (*L_eq-T_*) of construction. First, the noise-calculating models in consideration of noise synchronization and propagation as well as *L_eq-T_* are presented. Meanwhile, uncertainties, dynamics and interactions related to the *L_eq-T_* are discussed. Then, the simulation framework for implementing the noise-calculating models, including the modeling features for the noise-affected factors and the simulation strategy guiding simulation advancement, is proposed. Finally, an application study is presented to demonstrate and justify the proposed simulation method. 

## 2. Noise-Calculating Model Regarding Synchronization and Propagation

The noise sources in the construction process include various items of equipment as well as the noise-producing operations (e.g., blasting) that do not use any items of equipment. The noise may travel or propagate to the locations (called noise receptors) to disturb people. This section introduces the models for calculating the synchronized noise levels from multiple sources as well as the noise at the receptor by considering the noise attenuation during traveling or propagation.

### 2.1. Noise-Calculating Model Regarding Synchronization

Noise levels that can be heard by humans are commonly measured using a logarithmic scale named decibel A scale (dBA). The noise level for each item of equipment largely depends on the type or property and the quality or maintenance condition of the equipment. The noise levels for various categories of construction equipment used to be studied by Gilchrist et al. [15], Schexnayder and Ernzen [23], Thumann and Miller [24], Wilson [25] and Eaton [26].

During construction multiple items of equipment may be operated simultaneously to accomplish various construction activities. Hence the noise levels from various items of equipment need to be added or synchronized. The decibel A scale of the noise is not linear but logarithmic, so the noise levels cannot be added directly like real numbers. According to the acoustic theory [11], the noise level (*L*) is measured as follows:

(1) $$L=20\text{log}\frac{P}{P_{0}}$$

where *P* represents the sound pressure and *P*_0_ represents the reference sound pressure (i.e., 20 μPa). Summing or synchronization of the noise levels from *N* items of equipment can be achieved through Equation (2) [11]:

(2) $$\begin{array}{l} L_{S}=20\text{log}\frac{P_{S}}{P_{0}}=20\text{log}\frac{\left(P_{1}+P_{2}+\ldots +P_{N}\right)}{P_{0}}\\ =20\text{log}(10^{0.05L_{1}}+10^{0.05L_{2}}+\ldots +10^{0.05L_{N}})\\ =10\text{log}(\sum_{i=1}^{N}10^{0.1L_{i}}) \end{array}$$

The above synchronization equation can be expressed as:

(3) $$L_{s}=f_{s}(\bar{L_{s}})\begin{array}{cc} , & \bar{L_{s}}=\left\{L_{1},L_{2},\ldots ,L_{N}\right\} \end{array}$$

In contrast to the branch method [22] used to facilitate the synchronization calculation by hand, the above synchronization equations can be easily and efficiently implemented through a computer-based technique.

### 2.2. Noise-Calculating Model Regarding Propagation

In addition to negatively affecting the on-site worker, the noise will travel or propagate to the locations or receptors such as residences, office buildings, hospitals, and educational institutions. In consideration of noise attenuation during the traveling or propagation process, the noise level at the receptor(s) can be calculated as:

(4) $$L=f_{s}(\bar{L})\begin{array}{cc} , & \bar{L}=\left\{\left(L_{1}-A_{1}),(L_{2}-A_{2}),\ldots ,(L_{N}-A_{N}\right)\right\} \end{array}$$

where *f_s_* is the synchronization function as Equation (2), *L* is the resultant noise at the receptor, *L_i_* is the noise level from the *i*-th source (equipment) and *A_i_* is the attenuation of the noise from the *i*-th source during the propagation process. The attenuation of the noise is composed of the attenuations respectively due to geometrical divergence, air absorption, ground absorption and reflection [25], which can be expressed as:

(5) $$A_{i}=A_{i,div}+A_{i,air}+A_{i,gro}+A_{i,ref},\begin{array}{c} i=1,\ldots ,N \end{array}$$

The noise attenuation due to geometrical divergence (*A_i,div_*) is calculated as:

(6) $$A_{i,div}=20\text{log}(r_{i,1}/r_{i,2}),\begin{array}{c} i=1,\ldots ,N \end{array}$$

where *r_i,_*_2_ is the distance between the *i*-th noise source and the receptor, and *r_i,_*_1_ is the calibration distance for the *i*-th noise source, which is generally assumed to be 15.2 m [27].

The air attenuation due to air absorption is quite small except for very high frequencies and can be neglected at short distances less than several hundred meters [15]. Therefore the air absorption *A_i,air_* regarding the noise at the receptor less than several hundred meters away from the construction site is negligible.

The ground absorption (*A_i,gro_*) is related to the hardness of the ground surface and the distance traveled by the noise. With regards to predicting the construction noise, ground absorption can be simply calculated through the following equation [27]:

(7) $$A_{i,gro}=4.8-(2h_{i,m}/r_{i,2})(17+300/r_{i,2})\begin{array}{cc} , & i=1,\ldots ,N \end{array}$$

where *h_i,m_* is the mean height (in meters) of the propagation path for the noise from the *i*-th source, and *r*_*i*,2_ is the distance (meters) between the *i*-th noise source and the receptor. The attenuation due to reflection (*A*_*i*,ref_) is correlated to the height (*h_i,s_*) of the *i*-th noise source and the receptor height (*h_r_*) and can be calculated based on the following equation [27]:

(8) $$A_{i,ref}=-0.0053\times {\left(h_{r}/h_{i,s}\right)}^{4}+0.12\times {\left(h_{r}/h_{i,s}\right)}^{3}-1.1596\times {\left(h_{r}/h_{i,s}\right)}^{2}-6.4484\begin{array}{cc} , & i=1,\ldots ,N \end{array}$$

## 3. Noise-Calculating Model for Equivalent Continuous Level 

The construction process is a representative case where the noise levels fluctuate from time to time because different types or items of equipment may be operated simultaneously and stopped or restarted according to schedules or logical dependencies. Therefore, ISO [11] recommended converting the instantaneous noise level to a single number called “equivalent continuous noise”, i.e., *L_eq-T_*, to describe the noise level over a period of time *T*. The *L_eq-T_* is defined as the average-weighted sound pressure level of a noise fluctuating over a period of time *T*, and is calculated as:

(9) $$L_{eq-T}=10\text{log}\left[\frac{1}{T}\int_{0}^{T}10^{0.1L}dt\right]$$

For the discrete cases where noise levels may change at discrete points of time but remain constant between two adjacent discrete points of time, the *L_eq-T_* can be calculated as:

(10) $$L_{eq-T}=10\text{log}\left[\frac{1}{T}(10^{0.1L_{1}}T_{1}+10^{0.1L_{2}}T_{2}+,\ldots ,+10^{0.1L_{n}}T_{n})\right]$$

where *T* (=*T*_1_ + *T*_2_ +, …, + *T_n_*) is the period of time corresponding to the *L_eq-T_*, *T*_i_ (*i* = 1, ..., *n*) is a shorter period of time divided from *T*, and *L*_i_ is the assumed constant noise level over the period of time *T*_i_. The construction process can be considered as the case in which the noise level changes at the discrete points of time when an item of equipment is started or stopped from time to time and the noise level remains constant during the operation period once it is randomly determined for each cycle. Consequently the *L_eq-T_* for the construction noise can be calculated using Equation (10).

**Figure 1:**
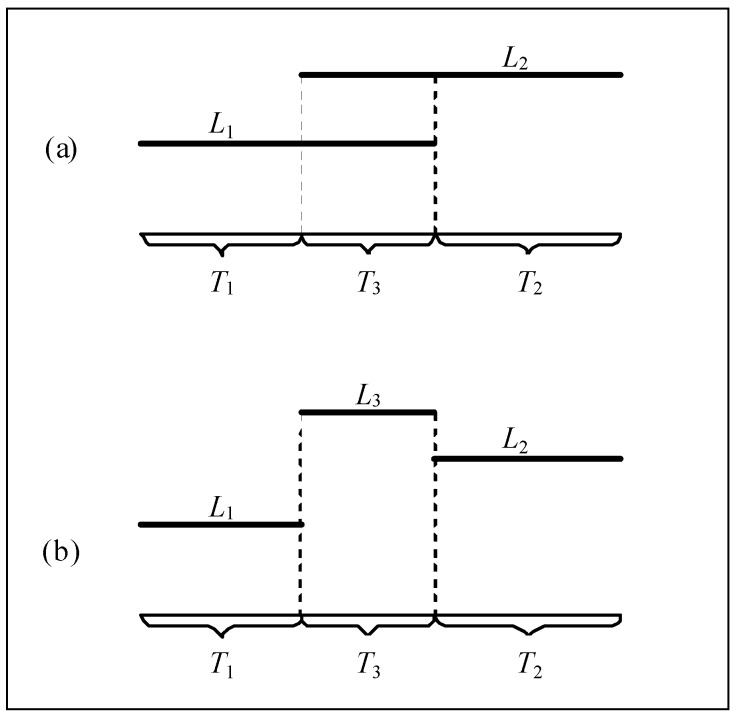
Illustration of overlap of multiple noises. (**a**) Before summing for *T*_3_; (**b**) After summing for *T*_3_.

Multiple items of equipment serving different construction activities may be operated simultaneously during some periods of time. For example, two items of equipment that respectively serve different activities and produce the noise levels *L*_1_ and *L*_2_ are operated simultaneously during the period of *T*_3_ as shown in Figure 1a, though they are started and stopped at different times. To calculate the *L_eq-T_* over the period of *T* (=*T*_1_ + *T*_2_ + *T*_3_) at the receptor, the overlapped noises during the period of *T*_3_ should be synchronized using Equation (2) or Equation (3) to get the noise level *L*_3_ at first, then Equations from (4) to (8) are used to calculate the noise levels *L*_1_, *L*_2_ and *L*_3_ (see Figure 1b) at the receptor by accounting for the attenuation during propagation. Finally the noise levels *L*_1_, *L*_2_ and *L*_3_ at the receptor, which correspond respectively to the periods of *T*_1_, *T*_2_ and *T*_3_, are average-weighted to obtain the *L_eq-T_* using Equation (10).

## 4. Uncertainties, Dynamics and Interactions Related to Equivalent Continuous Noise

The noise level depends on the properties and maintenance conditions of equipment as well as the on-site conditions, which are uncertain for different cases, so the noise level at the source may be uncertain or random. The type and age of an item of equipment should influence the equipment’s properties. The geometric locations or layouts of the equipment and the receptor, such as heights and distance of/between the noise source and the receptor, also affect the noise propagation and the noise level at the receptor [27]. The geometric locations may be uncertain majorly due to the temporary and dynamic natures in the construction process, thus dynamically influencing the noise level at the receptor according to Equations from (2) to (8). The uncertain factors can be modeled in terms of probability distributions with respect to different cases.

In addition to equipment properties and geometric parameters which greatly affect source noise levels, the *L_eq-T_* also relies on the proportion of time during which multiple items of equipment operate concurrently and the number of concurrently operated equipment. Meanwhile, the proportion of time and the number of concurrently operated equipment are affected by the start time, duration and preceding-logical conditions and resource requirements of each activity. Moreover, these factors affecting the *L_eq-T_* may be stochastic and interact with each other. It is also indicated that the *L_eq-T_* varies dynamically over time because of the proportion of time and the number of concurrently operated equipment change during the construction process.

Though the general methods including the Monte Carlo methods [15,16] could address uncertainties or randomness, but they could not model the dynamics and complex interactions over time. The discrete-event simulation (DES) method has the capabilities for modeling uncertainties, dynamics and interactions [28], so the DES method is selected to estimate the construction noise in terms of *L_eq-T_* by implementing the noise-calculating models from Equations (2)–(10) as well as accounting for the uncertainties, dynamics and complex interactions.

## 5. Simulation Framework for Implementing the Noise-Calculating Models

A DES platform, i.e., the activity object-oriented simulation (AOOS) [20], is modified to implement the noise-calculating models described above for predicting the construction noise in terms of *L_eq-T_*. The simulation framework regarding the modeling features and simulation strategy needs to be modified.

### 5.1. Modeling Features for the Noise-Affected Factors 

The activity object-oriented simulation (AOOS) [20] was developed based on activity cycle diagram (ACD) [28] and object-oriented approach. The AOOS adopts the simulation model similar to the activity-on-node (AON) network to describe a construction process over a computer-based platform, on which simulation experimentation is performed. Unlike CYCLONE [18] and STROBOSCOPE [21] that use two kinds of modeling elements for activities (i.e., conditional and unconditional activities) and queue elements for entities flow, the AOOS model does not classify the conditional and unconditional activities and does not consider the queue elements.

In view of the requirements to model the noise-affected factors for implementing *L_eq-T_* calculation through simulation as well as the particularities of the AOOS model, the AOOS model should be developed according to the following modeling procedure:

1. Decompose a construction process into a series of activities, with random activity durations in terms of probability distributions and the logical dependencies among the activities;
2. Identify the number of each resource including items of equipment required by each activity, including whether the noise from the equipment at the current activity is considered according to the distance of the current activity to the receptor;
3. Determine overall properties and total numbers of all types of equipment required during the construction process, including the uncertain source noise level of each item of equipment in terms of probability distribution;
4. Determine the distance between the noise source (on-site equipment) and the receptors (neighboring points) as well as the noise source height and the receptor height;
5. Determine the fixed period of time *T* and the interval between two adjacent periods of time to calculate the *L_eq-T_* during the simulation experiments.

**Figure 2:**
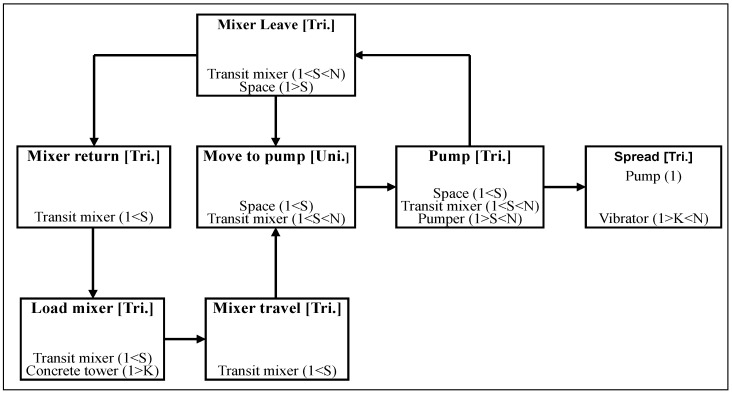
An AOOS model for predicting the noise from a foundation concreting project.

Figure 2 is an AOOS model for predicting the noise from a foundation concreting project, where seven activities are decomposed according to the characteristics of the concreting process. The name of each activity is displayed at the top line of the activity element. Three types of information, such as activity name followed by the probability distribution type for the random activity duration, e.g., triangular, uniform, normal, beta and exponential distributions which are respectively represented by “Tri.”, “Uni.”, “Nor.”, “Bet.” and “Exp.”, logical dependency on the preceding activity and resource-related information, are listed in the activity element from top to bottom. 

If there are the same resources between the current activity and another one, the logical dependency is indicated by an arrow line rather than the logical information indicated in the activity element. Various types of resources (including equipment generating emissions and noise) are displayed line by line, and each line of the resource-related information includes the resource name, resource’s flowing direction indication (“K” for remain, “S” for flowing to one succeeding activity and “F” for flowing to the activity meeting the conditions earliest), and the number of this type of resource. If the resource is an item of equipment producing noise and the current activity (or location) is close to the receptors and should be controlled for noise, each line of the resource-related information also includes the noise-producing indication “N” after the flowing direction indication in turn. In Figure 2, for example, the noises from the transit mixer, concrete pump and vibrator that are operated at the activities “Move to pump”, “Pump”, “Spread” and “Mixer leave” are considered because they are close to the receptors sensitive to the noise. Other three activities “Mixer travel”, “Mixer return”, and “Load mixer” do not happen completely at construction site and are somewhat far away from the receptors, hence the noises from these activities are not considered. It is noted that the noises from the operations that do not happen completely at construction site are not considered in the simulation method. The modeling steps 1 and 2 are achieved through the activity-associated dialog. The modeling steps 3–5 that concern the equipment-configuration, noise-affecting factors such as the source noise level of each item of equipment in terms of probability distribution and relevant parameters for calculating *L_eq-T_* are achieved through an overall dialog. The probability distribution types considered for the random source noise levels contain the triangular, uniform and normal distributions. The requirements on the resource-attributes (e.g., empty or full of a truck) for each activity, which reflects the starting sequence of the activities, are inputted and displayed through the activity-associated dialog. Detailed descriptions of the AOOS model are not presented herein due to length limitation of the paper and readers can refer to Zhang et al. [20].

### 5.2. Simulation Strategy Incorporated with the Noise-Calculating Models

In order to achieve simulation experimentation based on the built model, a simulation strategy is required to advance simulation clock and timely control various operations such as timely tracing noise variation and calculating noise level at discrete points of time such as start and end events [28]. Like the three-phase strategy for the CYCLONE [18] modified based on the activity scanning (AS) strategy, the AOOS strategy [20] is also modified based on the AS strategy in accordance with the AOOS model. Because the simulation under study is developed by utilizing the AOOS, the AOOS strategy is used herein. Before advancing the simulation time, the start conditions are checked so that the start events meeting the conditions are initiated, which is performed continuously until no activities meet the conditions. The start time of an activity should be the latest available time of the required resources and the logical dependencies. 

**Figure 3:**
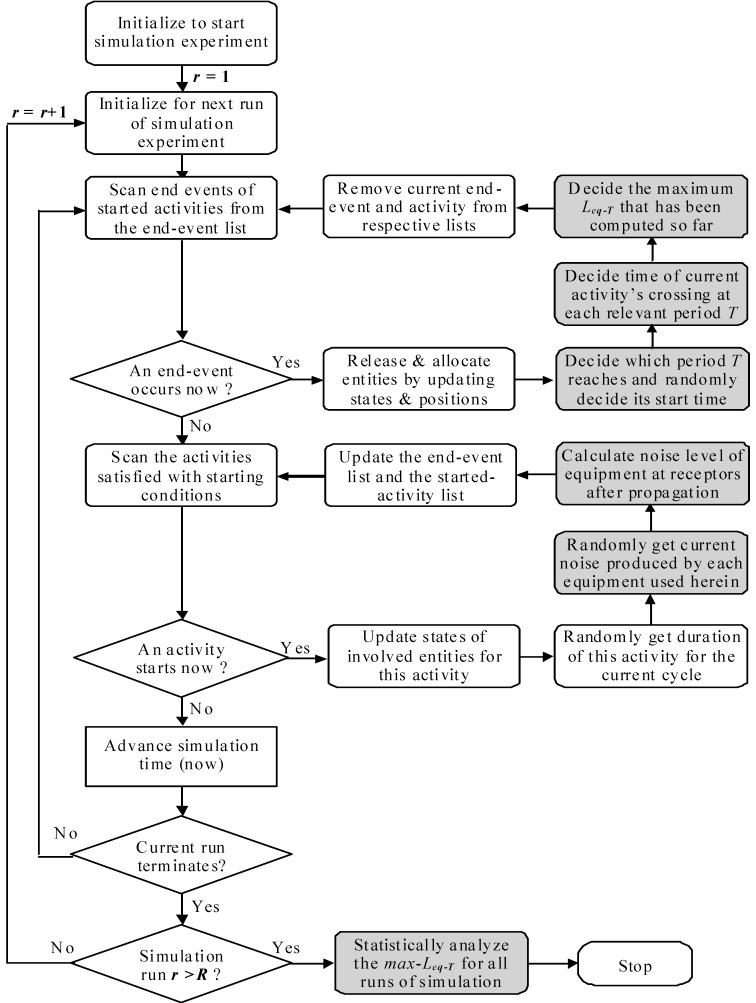
Flowchart of the simulation strategy incorporated with the noise-calculating models.

Figure 3 shows the flow chart of the improved AOOS strategy, where the parts marked in dark rectangules are added based on the noise-calculating models including Equations from (2) to (10). The noise level for each piece of equipment at each activity may be random and may change upon the instantaneous occurrence of a start or end event. Once a start event is activated to start an activity, the real-time noise level of a piece of equipment at an activity for current cycle must be determined randomly according to the probability distributions given in modeling. For detailed descriptions of the AOOS strategy, we refer to Zhang et al. [20].

**Figure 4:**
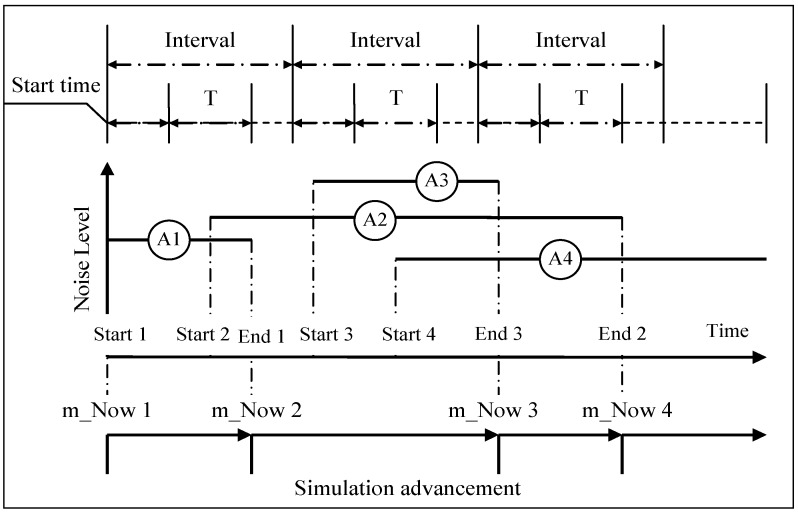
Illustration of each period of time *T* for calculating *L_eq-T_* along with simulation advancement.

The procedure of calculating the *L_eq-T_* during the simulation experiment is illustrated in Figure 4, where four activities (i.e., A1, A2, A3 and A4) are involved and the *L_eq-T_* over the period of time *T* is timely calculated during the simulation experiment. In accordance with the measurement procedure, the *L_eq-T_* is repetitively calculated for each equal interval of the construction duration. The period of time *T* and the interval between two adjacent periods of time which are assumed constant across the simulation experiment need to be specified before simulation. The start time to calculate the *L_eq-T_* at each interval is determined randomly from 0 to interval subtracting the time *T*.

Each period of *T* should be automatically advanced with the simulation progress, and the start time for each period of *T* should be randomly determined. The start and end events associated with each activity should be checked to see which periods of *T* the current activity crosses when initiating the end event. Then the noise level associated with the activity and the time of the activity crossing in this period of *T* can be timely determined, thus computing the time-weighted noise level. The time-weighted noise level related to each activity should be added to calculate the *L_eq-T_* for the certain period of *T* according to Equation (10). 

The value of *L_eq-T_* for each period may be obviously different due to dynamic natures and long construction duration, so the maximum value of the *L_eq-T_* from a lot of periods, that is, *max*-*L_eq-T_*, should be determined. One *max*-*L_eq-T_* can be obtained for each simulation run, so a series of the *max*-*L_eq-T_* samples can be collected after simulation and the statistic results (e.g., confidence interval) of the *max*-*L_eq-T_* can be calculated.

According to the enhanced AOOS modeling features and the modified AOOS strategy, visual C++ that was used to develop the prototype of the AOOS system is also used to implement the proposed DES method. 

## 6. Application Study

The proposed DES method is illustrated through predicting the *L_eq-20m_* for a foundation concreting project. The DES method is also verified and validated through comparing the simulation results with the measurement results.

### 6.1. A Foundation Concreting Process and Simulation Model

**Figure 5:**
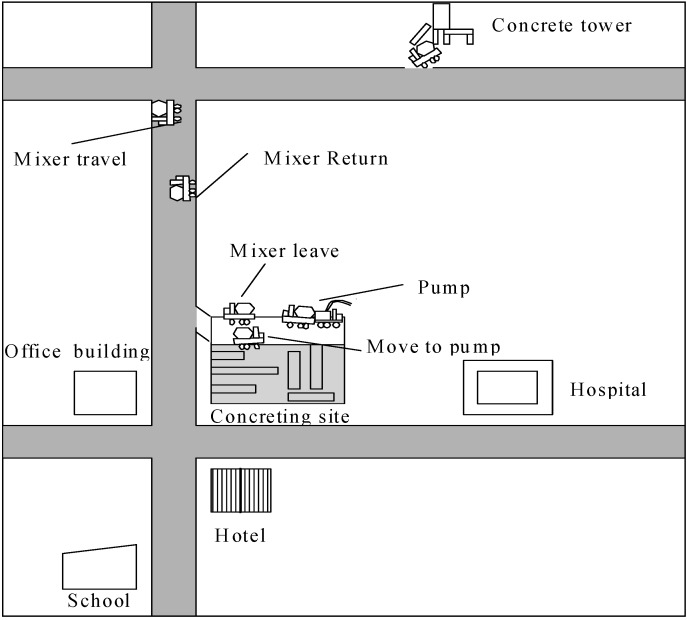
Layout of the foundation concreting project.

This foundation concreting project of 1440 m^3^ is for a superstore construction located in the downtown area of Chenngdu (Figure 5).

The concreting project was achieved through pumping concrete transported from a concrete plant. Six transit mixers, one concrete pump and one vibrator are used to perform the concreting. The neighboring receptors that are sensitive to the noise include an office building, a school, a hotel and a hospital. The equipment-configuration including the properties and source noise levels are given in Table 1, where the uncertain source noise levels of equipment regarding maintenance conditions and location changes are described through the summarized probability distributions for the equipment used [15]. Note that the source noise level of the transit mixer is different when the transit is loaded or empty. The concreting process consisting of transporting, pumping and vibrating produces the noise annoying the neighboring office building, school, hotel and hospital. The distances between the doors of the four receptors and the concreting site are respectively 32 m, 95 m, 41 m and 147 m. The average height of the noise source for the concreting site where each item of equipment operates is 1.25 m, while the heights of the four receptors are specified to be 1.3 m as the same height for field measurement described below. 

The activity durations are dependent of the properties of equipment and on-site conditions, and are usually represented through probability distributions. The triangular distribution is regarded a simplistic and effective description of an input variable (namely input modeling) for DES if the minimum, most-likely, and maximim of the input variable can be estimated through field measurement or knowledg and experience [28]. For this case study 80 samples of the duration for each activity have been collected. Then the distribution of the frequency that an activity duration falls into each of the 10 intervals between the minimum and maximum of the samples can be obtained. Based on the frequency distribution (or histogram) of the duration samples, the minimum, most-likely, and maximim values of the duration for each activity were specified, and then the triangular distribution is determined to describe the activity duration. If the range between the minimum and maximum values of the duration samples for an activity was less than 1.0 min, the uniform distribution is used to describe the random activity duration. For example, the duration of the activity “Mixer travel” has the the minimum 9.0 and maximum 12.0 respectively, and most of the samples fall into the interval [10,11] with the average of 10.5, then the tranigular (Tri.) distribution (9,10.5,12) is used to describe the random duration of “Mixer travel”. For the activity “Move to pump”, the range between the minimum 1.0 and maximum 1.2 of the samples is less than 1.0 min, a uniform (Uni.) (1,1.2) is used to describe the random duration of “Move to pump”. In addition, chi-squared and Kolmogorov-Smirnov tests have been performed to evaluate if the distributions appropriately represent the activity durations. The *p*-values from the two kinds of tests on each distribution are greater than 0.05, so the triangular or uniform distributions are appropriate for representing the activity durations because the *p*-value greater than 0.05 is considered acceptabe for DES input modeling [29].

The DES model, i.e., AOOS model, for modeling the foundation concreting project is developed as described in the previous section and shown in Figure 2. The random activity durations in terms of probability distributions shown in Table 2 need to be inputted during modeling. Note that the space at the concreting site is enough to park two trucks, including one being pumped and the other waiting for pumping. The description about the AOOS model has been stated in the former section of the paper.

### 6.2. Simulation Experiments and Result Analysis

In order to evaluate the noise level with respect to the noise standard China GB12523 [10] in terms of *L_eq-20m_*, the period of 20 min is selected for the simulation experiments. According to the capacities of the transit mixer and concrete pump as well as the concreting volume of 1440 m^3^ to be completed, each simulation run is performed until the concreting task is completed. The *L_eq-20m_* is continuously calculated under the interval of 60 min and the start time of measurement is randomly determined from 0 to 40 min at each period of 60 min. The mean duration of the concreting project is 1501.03 min (i.e., 25.02 h) with standard deviation 26.35. It means that the *max-L_eq-20m_* is selected among 25 pieces of the *L_eq-20m_* data for each simulation run. To obtain statistically enough samples, 100 runs of simulation experiments (i.e., a fixed simulation ending criterion) are performed, so 100 pieces of the *max-L_eq-20m_* data are obtained and the statistic results can be calculated. Table 3 lists the simulation results including the 90% confidence intervals and mean values of the *max-L_eq-20m_* for the four receptors near the foundation concreting site.

According to the construction noise standard China GB12523 [10], the *max*-*L_eq-20m_* should not exceed 70 dBA and 55 dBA for daytime and nighttime, respectively. Table 3 shows that the noise levels at the office building, school and hotel all exceed the noise standards. Though the noise level at the hospital does not exceed the daytime standard, it still exceeds the noise standard II of China GB3096 [30] with respect to urban area (60 dBA for daytime and 50 dBA for nighttime). It is demonstrated that the simulation method is able to predict the *L_eq-T_* at the planning stage, thus evaluating whether the noise level meets the noise standard. 

The noise level or *max-L_eq-T_* can be affected by not only the equipment-configurations and geometric conditions but also the duration, preceding-logical conditions and resource requirements of each activity, which may affect start or end times and concurrent operation periods of multiple items of equipment as shown in Figure 4. Hence the construction plans concerning these factors can be appropriately adjusted so as to reduce the noise level according to the noise standard. For example, when the items of equipment have the source noise levels lower than those in Table 1, the noise levels (*max-L_eq-T_*) at the four receptors will be lower than the values in Table 3 if other parameters such as activity durations are the same. If the activity durations are different from those in Table 2, the resultant noise levels at the four receptors will also be affected, which should be estimated using the methods like the proposed DES. The proposed DES method provides a platform to test various construction plans and help decide which construction plan is the most appropriate with regards to the *L_eq-T_*.

### 6.3. Comparison between Simulation and Measurement Results

To verify and validate the simulation results, field measurement of the *max-L_eq-20m_* for the four neighboring receptors had been performed during the concreting process. The field measurement was accomplished using two pieces of noise measuring instruments, i.e., a noise statistics analyzer AWA6218A made in China. This kind of noise measuring instrument is a highly intelligent pocket noise measuring instrument and is capable of directly measuring the *L_eq-T_* and performing measurement automatically and repetitively from hour to hour. Its performance has been certified in line with the standard such as GB/T17181-1997 issued by China and IEC61672-2002-2 released by the International Electrotechnical Commission (IEC) [31]. Due to availability of only two pieces of the measuring instruments, the manual measuring method rather than automatic measuring method was adopted, consequently the *L_eq-20m_* at each receptor can be measured only once per hour. Each measuring instrument was allocated to measure two receptors and moved continuously among the two receptors. The four measurement points were selected at the places just outside the doors of the four receptors and 1.3 m high, meeting the measuring stipulation of China GB12523 [10]. As a result, 25 pieces of the *L_eq-20m_* data had been collected for each of the four receptors during the concreting project of around 25 h, and then the *max-L_eq-20m_* had been determined.

The background noise (in terms of equivalent continuous noise over 20 min) during which the concreting operation was not performed had been also investigated. In addition, the difference of the background noise between rush hour and non-rush hour was also considered. The average levels (namely *aveBk-L_eq-20m_*) at the office building, hotel, school and hospital are 72.2 dBA, 72.2 dBA, 62.9 dBA and 62.3 dBA respectively. Note that the background noises at the receptors close to the crossroad are relatively high. According to Equation (2) calculating the summed noise level of multiple sources, the measured *max-L_eq-20m_* should be adjusted based on the following equation by ignoring the background noise:

(11) $$\text{max}-L_{eq-20m}=10\text{log}(10^{0.1\text{max}-L_{eq-20m}}-10^{0.1aveBk-L_{eq-20m}})$$

According to the measured noise levels and Equation (11) for adjustment, the final measured *max-L_eq-20m_* are obtained as listed in Table 3.

Table 3 lists the simulation results (e.g., *max*-*L_eq-20m_*) in terms of the mean values and 90% confidence intervals as well as the measurement results. It is shown that the measurement results or *max*-*L_eq-20m_* for the office building, hotel, school and hospital are in the ranges of 90% confidence interval of the simulation results and are very close to the mean values of the simulation results. The comparison between simulation and measurement results demonstrates that the proposed simulation methodology is able to provide effective estimation of the *L_eq-T_*.

## 7. Conclusions

This paper presents a discrete-event simulation method for predicting construction noise in terms of equivalent continuous level (*L_eq-T_*) by enhancing the relevant simulation strategy with incorporation of noise-calculation models. The proposed method is able to consider uncertainties, dynamics and interactions during construction in predicting the *L_eq-T_*. The *L_eq-T_* obtained through the simulation method is more meaningful than the instantaneous noise level of complex construction environments. The simulation method is more practical than field measurements to estimate construction noises, especially during the planning period, and is capable of evaluating construction plans to help make decisions to address noise levels in terms of *L_eq-T_*. There exist some limitations in the proposed simulation method, such as inability of considering the noise barriers or buildings between the source and receptors, including sound reverberation from the neighboring buildings. Further studies will focus on these issues.

## Figures and Tables

**Table 1:** Properties and source noise levels of various types of equipment for concerting.

Category	Mode	Country of Manufacture & Year	Capacity/Power	Number	Noise (dBA)
Transit mixer	CA5250GJBP66K2L1T1E4	China, 2005	4.9 m^3^	6	Uniform (85, 88) when loaded
					Uniform (80, 85) when empty
Concrete pump	HBT60.13-90S	China, 2001	60 m^3^/h	1	Uniform (82, 85)
Vibrator	ZN-90	China, 2001	2.2 kW	1	Uniform (76, 80)

**Table 2:** Activity durations (min) in terms of probability distributions.

Activity	Load Mixer	Mixer Travel	Move to Pump	Pump	Mixer Lave	Spread	Mixer Return
Duration	Tri. (2.5, 3.0, 3.5)	Tri. (9, 10.5, 12)	Uni. (1, 1.2)	Tri. (4.5, 5, 5.5)	Uni. (0.8, 1)	Tri. (4.5, 5, 5.5)	Tri. (8, 9, 10)

**Table 3:** Comparison between simulation results and measurement results.

Receptor	Distance to Source (m)	Simulation Results (dBA)		Measurement Results (*max-L_eq-20m_*) (dBA)
		90% Confidence Interval	Mean *max-L_eq-20m_*	
Office Building	32	(86.41, 87.85)	87.07	87.02
Hotel	41	(83.27, 84.30)	83.85	83.99
School	95	(74.13, 74.92)	74.55	74.25
Hospital	147	(70.45, 71.32)	70.89	70.58
